# Draft Genome Sequence of *Bacillus* sp. Strain SB47, an Obligate Extreme Halophile Isolated from a Salt Pan of the Little Rann of Kutch, India

**DOI:** 10.1128/genomeA.00816-13

**Published:** 2013-10-10

**Authors:** Kamal Krishna Pal, Rinku Dey, Manesh Thomas, Dharmesh Sherathia, Trupti Dalsania, Ilaxi Patel, Kinjal Savsani, Sucheta Ghorai, Sejal Vanpariya, Bhoomika Sukhadiya, Mona Mandaliya, Rupal Rupapara, Priya Rawal, Anil Kumar Saxena

**Affiliations:** Microbiology Section, Directorate of Groundnut Research, Junagadh, Gujarat, Indiaa; Division of Microbiology, Indian Agricultural Research Institute, New Delhi, Indiab

## Abstract

Here, we report the 4.46-Mbp draft genome sequence of *Bacillus* sp. strain SB47, an extreme halophile isolated from a salt pan of the Little Rann of Kutch, India. Exploring the genome of this organism will facilitate the understanding and isolation of the gene(s) involved in its extreme osmotolerance.

## GENOME ANNOUNCEMENT

*Bacillus* sp. strain SB47, an obligate extreme halophile and endospore-forming bacterium, was isolated from a salt pan of the Little Rann of Kutch, India. It grows optimally at a 15% NaCl (range, 5 to 35%) concentration in medium at 37°C and at pH 7.5. The genome of *Bacillus* sp. strain SB47 was sequenced to understand the mechanisms of its extreme osmotolerance and to isolate the relevant gene(s).

The whole genome of *Bacillus* sp. SB47 (G+C content of 45.50%) was sequenced using the Roche 454 Genome Sequencer (GS FLX) at Macrogen Inc., South Korea, through Sequencher Tech Pvt. Ltd., Ahmedabad, India, by both shotgun and mate-paired library sequencing. In shotgun sequencing, an average read length of 360 bp was generated from 603,934 reads of 217,996,405 bases. Sequencing of the mate-pair libraries gave 151,827 and 131,939 reads, respectively, with average read lengths of 475 bp and 455 bp, respectively.

*De novo* assembly was performed using the GS *de novo* Assembler version 2.6 (1) with approximately 76-fold coverage, and 10 scaffolds of 4,468,918 bp and 33 scaffold contigs of 4,464,274 bp with average lengths of 446,891 bp and 135,281 bp, respectively, were obtained. An N_50_ scaffold length of 2,486,969 bp (4,808 bp and 2,486,969 bp for the smallest and largest scaffolds, respectively) was obtained. Similarly, an N_50_ contig length of 231,087 bp (1,620 bp and 807,418 bp for the smallest and largest contigs, respectively) was obtained. All assembly data were deposited in the DDBJ/EMBL/GenBank nucleotide sequence database.

The draft genome sequence was annotated by the RAST server (2), Glimmer 3 (3, 4), GeneMark (5, 6), the KEGG database (7), tRNAScan-SE (8), RNAmmer (9), and Signal P4.1 (10).

Using the different softwares, we predicted 4,718 coding sequences (CDSs), with 3,901,977 bp in the CDSs. There were 74 RNA-encoding genes (68 tRNA, 6 rRNA) and 396 subsystems. Among the CDSs, 2,650 are not in a subsystem (1,047 nonhypothetical CDSs, 1,603 hypothetical CDSs), whereas 2,068 CDSs (1,929 nonhypothetical, 139 hypothetical) are in a subsystem. RAST annotation also revealed the association of 105 genes involved in stress responses in this organism: 10 in osmotic stress (1 in osmoregulation, 9 in choline and betaine uptake and betaine biosynthesis), 46 in oxidative stress (7 in protection from reactive oxygen species [ROS], 28 in oxidative stress, 1 in NADPH:quinine oxidoreductase 2, 1 in glutathione:nonredox reactions, 6 in redox-dependent regulation of nucleus processes, and 3 in glutaredoxins), 1 in cold shock, 16 in heat shock, 10 in detoxification, 1 in periplasmic stress, and 21 in no subcategory, with 237 signal peptides. Similarly, 2,186 CDSs were mapped to different biochemical pathways of KEGG (K00003 to K16706). The genes responsible for the production of different enzymes for the biosynthesis of valine, leucine, and isoleucine (map00290) and a number of genes involved in ABC transporters (map02010), including transporters for alkanesulfonate (SsuA, SsuC), glycine betaine/proline (ProX, ProW, ProV), osmoprotectants (OpuBC, OpuBB, OpuBA), and phosphate transporters (PstA, PstB, PstC, PstS), were also mapped. Similarly, genes for two-component systems (map02020), like those involved in the response to K^+^ limitation and K^+^ transport (KdpD, KdpA, KdpB, KpdC) and genes for salt stress degradative enzymes (DegS, DegU), have been mapped.

Deciphering the genome of this organism further will facilitate the understanding of obligate and extreme halophilism and the genes, biochemical pathways, and metabolites involved in osmotolerance.

### Nucleotide sequence accession numbers.

This whole-genome shotgun project has been deposited at DDBJ/EMBL/GenBank under the accession no. ATNR00000000. The version described in this paper is version ATNR01000000.
